# Supplementary material for: Vagal sensory neurons mediate the Bezold–Jarisch reflex and induce syncope
Source: Nature. 2023 Nov 1;623(7986):387–96. doi: 10.1038/s41586-023-06680-7 (PMC10632149; doi:10.1038/s41586-023-06680-7)
Supplement: Supplementary file 1 — Reporting Summary [file 41586_2023_6680_MOESM1_ESM.pdf]

nature portfolio

Corresponding author(s): Vineet Augustine

Last updated by author(s): Sep 8, 2023

Reporting Summary

Nature Portfolio wishes to improve the reproducibility of the work that we publish. This form provides structure for consistency and transparency in reporting. For further information on Nature Portfolio policies, see our Editorial Policies and the Editorial Policy Checklist.

Statistics

For all statistical analyses, confirm that the following items are present in the figure legend, table legend, main text, or Methods section.

n/a	Confirmed	
☐	☒	The exact sample size (*n*) for each experimental group/condition, given as a discrete number and unit of measurement
☐	☒	A statement on whether measurements were taken from distinct samples or whether the same sample was measured repeatedly
☐	☒	The statistical test(s) used AND whether they are one- or two-sided<br>*Only common tests should be described solely by name; describe more complex techniques in the Methods section.*
☐	☒	A description of all covariates tested
☐	☒	A description of any assumptions or corrections, such as tests of normality and adjustment for multiple comparisons
☐	☒	A full description of the statistical parameters including central tendency (e.g. means) or other basic estimates (e.g. regression coefficient) AND variation (e.g. standard deviation) or associated estimates of uncertainty (e.g. confidence intervals)
☐	☒	For null hypothesis testing, the test statistic (e.g. *F*, *t*, *r*) with confidence intervals, effect sizes, degrees of freedom and *P* value noted<br>*Give P values as exact values whenever suitable.*
☒	☐	For Bayesian analysis, information on the choice of priors and Markov chain Monte Carlo settings
☒	☐	For hierarchical and complex designs, identification of the appropriate level for tests and full reporting of outcomes
☐	☒	Estimates of effect sizes (e.g. Cohen's *d*, Pearson's *r*), indicating how they were calculated

Our web collection on statistics for biologists contains articles on many of the points above.

Software and code

Policy information about availability of computer code

Data collection	FLUOVIEW 2.4.1.198, SmartSPIM GUI 2.1, SmartSPIM Destriping and Stitching 2.0, NIS-Elements 5.21.03, SpikeGLX 3.0, MoSeq2, AcqKnowledge, Vevo 3100, SpinView, CODA software 4.2 and custom Matlab 2022b, 2023a/ Python 3.6 code for collecting data.
Data analysis	Microsoft Excel, Premiere Pro CC 2022 (22.0), GraphPad Prism 9.4.1, 9.5.1 and 10.0.0, Imaris 9.2.1 and 10.0.0, ImageJ 1.8.0, Vevo LAB 5.5.0, AcqKnowledge 5.0, Kilosort3, Phy v2.0b1, SHARP-track, MoSeq2, Facemap, DeepLabCut 2.2, ToxTrac2.98, and custom R/Matlab 2022b, 2023a/ Python 3.6 code for analyzing behavior, Neuropixels and single cell RNA sequencing data (Seurat v3).

For manuscripts utilizing custom algorithms or software that are central to the research but not yet described in published literature, software must be made available to editors and reviewers. We strongly encourage code deposition in a community repository (e.g. GitHub). See the Nature Portfolio guidelines for submitting code & software for further information.

Data

Policy information about availability of data

All manuscripts must include a data availability statement. This statement should provide the following information, where applicable:

- Accession codes, unique identifiers, or web links for publicly available datasets
- A description of any restrictions on data availability
- For clinical datasets or third party data, please ensure that the statement adheres to our policy

All numerical data are included in Supplementary Information. All other data is large and available from the corresponding author upon reasonable request. SIngle cell sequencing data was obtained from GEO (GSE145216).

Human research participants

Policy information about studies involving human research participants and Sex and Gender in Research.

Reporting on sex and gender	NA
Population characteristics	NA
Recruitment	NA
Ethics oversight	NA

Note that full information on the approval of the study protocol must also be provided in the manuscript.

Field-specific reporting

Please select the one below that is the best fit for your research. If you are not sure, read the appropriate sections before making your selection.

☒ Life sciences ☐ Behavioural & social sciences ☐ Ecological, evolutionary & environmental sciences

For a reference copy of the document with all sections, see nature.com/documents/nr-reporting-summary-flat.pdf

Life sciences study design

All studies must disclose on these points even when the disclosure is negative.

Sample size	No sample size statistics was performed. Sample sizes were similar to recent papers (Augustine, V. et al. Nature 555, 204-209, 2018 and Lee, S. et al. Nature 568, 93-97, 2019).
Data exclusions	Animals with proper viral expression or implant placement were included in the analysis. For ECG and respiration, recordings with excessive noise were excluded. For facial features analysis, sessions where pupils were obscured were excluded.
Replication	All attempts at replication were successful. N numbers are indicated in the legends.
Randomization	No randomization was performed. Animals were arbitrarily assigned to experimental groups.
Blinding	No blinding procedures were performed. Representative data was based on at least 3 independent observations. Control and experimental groups were tested using similar conditions. Control and experimental data was analyzed using same criteria.

Reporting for specific materials, systems and methods

We require information from authors about some types of materials, experimental systems and methods used in many studies. Here, indicate whether each material, system or method listed is relevant to your study. If you are not sure if a list item applies to your research, read the appropriate section before selecting a response.

Materials & experimental systems

n/a	Involved in the study	
☐	☒	Antibodies
☒	☐	Eukaryotic cell lines
☒	☐	Palaeontology and archaeology
☐	☒	Animals and other organisms
☒	☐	Clinical data
☒	☐	Dual use research of concern

Methods

n/a	Involved in the study	
☒	☐	ChIP-seq
☒	☐	Flow cytometry
☒	☐	MRI-based neuroimaging

Antibodies

Antibodies used

Primary antibodies used were:
Chicken anti-GFP (ab13970, Abcam, 1:500); rabbit anti-RFP (600-401-379, Rockland, 1:500); rabbit anti-NPY2R (RA14112, Neuromics, 1:500); Alexa Fluor™ 647 conjugated GFP polyclonal antibody (A-31852, Thermo Fisher Scientific, 1:200).
Secondary antibodies used were:
Alexa Fluor® 647 AffiniPure Donkey Anti-Rabbit (711-605-152, Jackson ImmunoResearch, 1:500); Alexa Fluor® 488 AffiniPure Donkey Anti-Chicken (703-545-155, Jackson ImmunoResearch, 1:500); Cy™3 AffiniPure Donkey Anti-Rabbit IgG (H+L) (711-165-152, Jackson ImmunoResearch, 1:500).

Validation

All antibodies used were previously published, validated, and purchased from commercial vendors in the USA.
Chicken anti-GFP (ab13970, Abcam) has been referenced in at least 3182 publications according to vendor's website. From vendor's website: "Our GFP antibody does cross-react with the many fluorescent proteins that are derived from the jellyfish Aequorea victoria. These are all proteins that differ from the original GFP by just a few point mutations (EGFP, YFP, mVenus, CFP, BFP etc.)." Citation: Wang G et al. Somatic genetics analysis of sleep in adult mice. J Neurosci 42:5617-40 (2022). IHC; Mouse.
Rabbit anti-RFP (600-401-379, Rockland) has been referenced in at least 928 publications according to vendor's website. From vendor's website: "This product was prepared from monospecific antiserum by immunoaffinity chromatography using Red Fluorescent Protein (Discosoma) coupled to agarose beads followed by solid phase adsorption(s) to remove any unwanted reactivities. Expect reactivity against RFP and its variants: mCherry, tdTomato, mBanana, mOrange, mPlum, mOrange and mStrawberry. Assay by immunoelectrophoresis resulted in a single precipitin arc against anti-Rabbit Serum and purified and partially purified Red Fluorescent Protein (Discosoma). No reaction was observed against Human, Mouse or Rat serum proteins." Citation: Zhang C et al. A brainstem circuit for nausea suppression. Cell Rep. (2022) Applications: IHC, ICC, Histology; Mouse.
Rabbit anti-NPY2R (RA14112, Neuromics) has been referenced in at least 14 publications according to vendor's website. From vendor's website: "This NPY Y2 antibody has been extensively characterized in the spinal cord, brain, and distal colon." Citation: Neuropeptide Y in the Medial Habenula Alleviates Migraine-Like Behaviors through the Y1 Receptor. IHC; Mouse.
Alexa Fluor™ 647 conjugated GFP polyclonal antibody (A-31852, Thermo Fisher Scientific) has been referenced in at least 30 publications according to vendor's website. From vendor's website: "This Antibody was verified by Relative expression to ensure that the antibody binds to the antigen stated." Citation: Pyk2 suppresses contextual fear memory in an autophosphorylation-independent manner. IHC; Mouse.
Alexa Fluor® 647 AffiniPure Donkey Anti-Rabbit (711-605-152, Jackson ImmunoResearch) has been referenced in at least 434 publications according to vendor's website.
Alexa Fluor® 488 AffiniPure Donkey Anti-Chicken (703-545-155, Jackson ImmunoResearch) has been referenced in at least 546 publications according to vendor's website.
Alexa Fluor® 555 AffiniPure Donkey Anti-Rabbit (703-545-155, Jackson ImmunoResearch) has been referenced in at least 546 publications according to vendor's website.
Cy™3 AffiniPure Donkey Anti-Rabbit IgG (H+L) (711-165-152, Jackson ImmunoResearch) has been referenced in at least 1306 publications according to vendor's website.
We also validated all antibodies listed above with different tissues.

Animals and other research organisms

Policy information about studies involving animals; ARRIVE guidelines recommended for reporting animal research, and Sex and Gender in Research

Laboratory animals

Experiments were conducted on adult mice, both male and female between 1.5 - 6 months of age. The following mice lines were purchased from the Jackson Laboratory: C57BL/6J, stock number 000664; Ai9, stock number 007909; Ai32; stock number 012569; Slc17a6-Cre (also known as Vglut2-Cre), stock number 016963; Npy2r-ires-Cre, stock number 029285; Piezo2-EGFP-ires-Cre, stock number 027719. Mice were maintained in temperature (around 22-23C) controlled rooms with a 12 h:12h light:dark cycle (6am to 6 pm light on) and ad libitum access to chow and water.

Wild animals	No wild animals were used.
Reporting on sex	Both female and male mice were used.
Field-collected samples	No field-collected samples were used.
Ethics oversight	All procedures were done according to NIH and Institutional Animal Care and Use Committee (IACUC; protocol # 19-0018) guidelines at Scripps Research.

Note that full information on the approval of the study protocol must also be provided in the manuscript.
